# Relationship between Craniovertebral Abnormalities and Maxillary Lateral Incisors Agenesis: A Case-Control Study

**DOI:** 10.1155/2022/3389741

**Published:** 2022-09-06

**Authors:** Farhad Sobouti, Mehdi Aryana, Seyed Mohammad Ghadiri, Kiarash Modanloo, Sepideh Dadgar

**Affiliations:** ^1^Dental Research Center, Mazandaran University of Medical Sciences, Sari, Iran; ^2^Orthodontic Department, Dental Faculty, Mazandaran University of Medical Sciences, Sari, Iran; ^3^Student Research Committee, Dental Faculty, Mazandaran University of Medical Sciences, Sari, Iran; ^4^Dental Faculty, Shahid Beheshti University of Medical Sciences, Tehran, Iran

## Abstract

**Objectives:**

This study aimed to investigate whether the sella turcica bridging (STB) and ponticulus posticous (PP) are associated with the congenital missing maxillary lateral incisor (CMMLI), based on lateral cephalograms of patients who needed orthodontic treatment.

**Materials and Methods:**

This case-control study examined 160 panoramic images and lateral cephalograms of 2000 patients seeking orthodontic treatment. The case group included 80 patients with CMMLI (40 with unilateral and 40 with bilateral CMMLI) and the control group included 80 patients without CMMLI. Panoramic images were used to diagnose CMMLI and lateral cephalograms showed STB and PP extension. The researchers used statistical analyses to examine the relationship among STB, PP, and CMMLI (*P* < 0.05).

**Results:**

The prevalence of STB type I, II, and III was 47.5%, 35%, and 17.5% in the case group and 72.5%, 22.5%, and 5% in the control group, respectively, determining a positive relationship between CMMLI and STB and a significant relationship between bilateral CMMLI and STB (*P* < 0.05 for both). The prevalence of normal, incomplete, and complete PP extension was 80%, 5%, and 15% in the case group and 82.5%, 5%, and 12.5% in the control group, respectively. There was no significant relationship between CMMLI and PP extension (*P* > 0.05) and between the STB and the PP extension (*P* > 0.05).

**Conclusion:**

CMMLI was significantly related to STB but not to PP extension. Investigating the relationship between unilateral/bilateral CMMLI, STB, and PP has shown only a significant relationship between bilateral CMMLI and STB. There was no significant relationship between STB and PP extension.

## 1. Introduction

The sella turcica is located on the sphenoid's intracranial surface. Its anterior and posterior borders are represented by the tuberculum sella (TS) and the dorsum sellae (DS), forming a U-shaped structure that surrounds the pituitary gland. The distance between TS and DS is known as the sella length or interclinoid distance. The maximum distance between the head of TS and the furthest point on the inner surface of the posterior sella contour is called sella diameter [1, 2]. The “sella turcica bridging” (STB) morphologic variation (1.1% to 22%) is the consequence of the dura mater ossifying excessively between the anterior and posterior clinoid processes of the sphenoid, or a product of the sphenoid's improper embryologic development. Multiple craniofacial deformities and local dental anomalies, such as tooth transposition and congenitally missing teeth are thought to be associated with STB [3–5]. Ponticulus posticous (PP) or atlas arcuate foramen is defined as an abnormal bony protrusion arising from the superior articulating process of the atlas, partially or entirely surrounding the vertebral arteries and reaching the posterior arch of the atlas [6]. This anomaly can cause a variety of clinical issues such as headaches, dizziness, double vision, and soreness in the shoulder and neck are some of the clinical signs of PP [7].

Tooth agenesis is the most common congenital and developmental dental and craniofacial anomaly, usually resulting from disturbance at the early stages of odontogenesis [8–10]. Congenitally missing teeth are those caused by a germ lacking sufficient development to induce dental tissue differentiation, resulting in the missing of one or more teeth [11]. The etiology of tooth agenesis is unclear. However, a variety of probable explanations have been hypothesized, including inherited factors, trauma, localized inflammation, radiation, systemic diseases, and idiopathic diseases [12, 13]. After the third molars, the maxillary lateral incisors are the second most common case for agenesis. A number of findings have demonstrated the link between congenital missing maxillary lateral incisors (CMMLIs), displaced teeth, and premolar rotations. Patients with missing permanent teeth may experience various complications such as malocclusion, periodontal disease, lack of alveolar bone development, impaired chewing capacity, pronunciation issues, and alterations in skeletal relationships. The CMMLI depicts a clinical condition in which individuals have functional and aesthetic issues from an early age. Therefore, many patients with this anomaly frequently require orthodontic treatment or prosthetic tooth replacement [13, 14].

In orthodontics, the lateral cephalogram is the most commonly utilized diagnostic radiograph [3]. Although the cervical vertebrae maturation index is often employed to assess skeletal maturation and clarify the growth potential of young patients, not enough attention is devoted to the pathological aspect of the radiological anatomy of this region. A regular lateral cephalogram can detect significant diseases in the cranium and cervical spine, including the abnormal morphology of the sella turcica and the fusion of the cervical vertebrae, which is associated with craniofacial and dental anomalies [4, 15, 16].

Since STB is considered a developmental and genetic anomaly, the difference in the genetic composition of the populations may lead to different results [17]. In addition, the research conducted so far on the prevalence of STB in different patients in terms of age, race, skeletal classification, and anomalies has had contradictory results [18]. Moreover, few studies have been performed regarding the relationship between PP and dental anomalies. Therefore, this study aimed to identify a link between CMMLI, STB, and the development of PP, examining the lateral cephalograms of patients referred to an oral and maxillofacial radiology center in Iran.

## 2. Materials and Methods

The Research Ethics Committee of the Mazandaran University of Medical Sciences approved the study protocol (code: IR.MAZUMS.REC.1400.10433).

### 2.1. Subjects' Characteristics and Sample Size Determination

This case-control study was performed with 160 pretreatment lateral cephalograms and panoramic radiographs of two groups. The case group consisted of 80 radiographs of patients (40 patients with unilateral and 40 patients with bilateral CMMLI). The control group consisted of 80 radiographs of individuals who needed orthodontic treatment but did not have CMMLI. According to research conducted by Alqahtani [19], the sample size was determined to be 80 samples per group (case and control) (*p*1=46.9%, *p*2=69.4%, *α* = 0.05, and *β* = 0.2). The radiographs recorded between 2018 and 2021, belonged to patients seeking orthodontic treatment. The images were selected from among 2000 radiographs in the archive of a private oral and maxillofacial radiology clinic in Sari, Iran, under the supervision of an oral and maxillofacial radiologist.

The inclusion criteria encompassed were as follows:

1. Candidates for orthodontic treatment
2. Patients with unilateral or bilateral CMMLI for the case group
3. Patients with panoramic and cephalometric radiographs of high quality

Patients with the following conditions were excluded:

1. Systemic or syndromic disease
2. A history of chemotherapy or radiotherapy
3. A history of previous trauma
4. A history of orthodontic treatment or surgery

**Figure 1:**
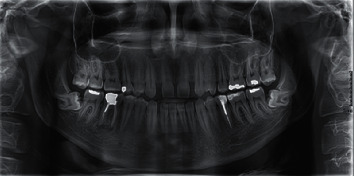
A panoramic radiograph of a patient with bilateral CMMLI.

Panoramic radiographs were used to diagnose the CMMLI (Figure 1). Lateral cephalograms were traced to assess the calcification of sella turcica. All panoramic radiographs and lateral cephalograms were obtained by an experienced technician using a Planmeca ProOne® panoramic device (PLANMECA OY©, Helsinki, Finland) and a Vatech lateral cephalometric device (PaX-i Insight model, Vatech Inc., NJ, USA) with a fixed magnification of 1.1. The lateral cephalograms were traced using the CephX (ORCA Dental AI, Las Vegas, NV) by a senior dental student under the guidance of an orthodontist [20].

### 2.2. Determining the STB and PP Extension

**Figure 2:**
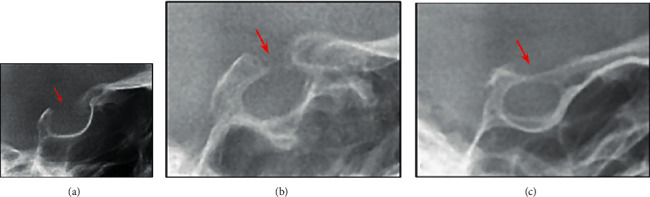
The classification of STB: (a) type I, (b) type II, and (c) type III.

According to Leonardi's conventional categorization [21], there are three varieties of STB based on the length and diameter of the sella (Figure 2):

- Type I or normal sella: the sella length is equal to or greater than ¾ of the sella diameter
- Type II or partial (incomplete) calcification: the sella length is equal to or less than ¾ of the sella diameter
- Type III or complete calcification: only the diaphragm of the sella is visible on the radiograph

**Figure 3:**
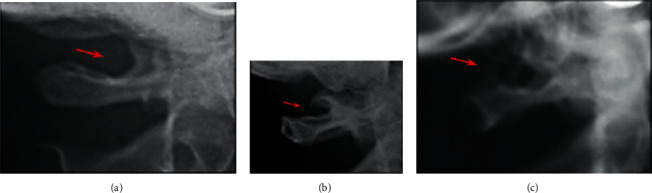
The classification of PP: (a) no bone emergence, (b) a partial bony emergence, and (c) a full bony bridge.

Complete (full bony bridge), incomplete (partial bony emergence), and normal (no bone emerging) were the three levels of PP development [6] (Figure 3).

### 2.3. Statistical Analysis

To describe the data, mean and standard deviation were used for quantitative variables and frequency percentages for qualitative variables. Chi-square and independent-sample *t*-test were used to compare the results of these groups. SPSS 25 software (IBM, Armonk, NY, USA) was used and the significance level was considered *P* < 0.05.

## 3. Results

Based on inclusion and exclusion criteria, 160 out of 2000 lateral cephalometric radiographs were selected and divided into case and control groups. The case group consisted of 80 patients with CMMLI, 40 with unilateral, and 40 with bilateral CMMLI. The control group included 80 patients with both maxillary lateral incisors present. The demographic features of the patients are presented in Table 1.

### 3.1. STB Measurements

**Figure 4:**
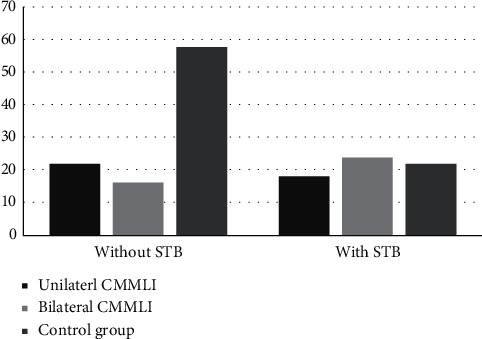
The prevalence of STB in case and control groups (*P*=0.06 for unilateral and 0.01 for bilateral compared to the control group).

The prevalence of STB was evaluated in the case and control groups.  Table 2 shows the frequency of each type of STB. According to this table, the calcification of interclinoid ligament (type II and III STB) was more prevalent in the patients with CMMLI compared to the control group (52.5% vs. 27.5%). The analysis performed using the Chi-square test has shown a significant difference (*P*=0.01), indicating a significant relationship between the occurrence of CMMLI and STB. Furthermore, the frequency of STB in patients with unilateral and bilateral CMMLI is shown separately in Table 2. As the data suggests, it is established that the prevalence of STB in bilateral CMMLI is more than in unilateral CMMLI. According to the performed analyses and comparisons, there was a statistically significant relationship between STB and the bilateral CMMLI (*P*=0.01) but no significant relationship was found between STB and the unilateral CMMLI (*P*=0.06) (Figure 4).

### 3.2. PP Measurements

**Figure 5:**
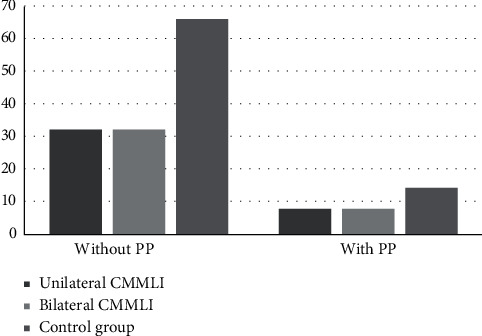
The prevalence of PP in case and control groups (*P*=0.12 for both unilateral and bilateral compared to the control group).

The frequency of PP was also measured in the case and control groups (Table 2). In the case group, 80% of patients were without and 20% with the PP extension (incomplete and complete). In the control group, 82.5% of patients were without and 17.5% with the PP extension. The Chi-square analysis found no statistically significant relationship between the PP extension and the CMMLI occurrence (*P*=0.12). The frequency of PP extension was also compared between unilateral/bilateral CMMLI groups and the control groups. The prevalence of PP extension was similar in unilateral and bilateral CMMLI groups. The analysis has shown no significant relationship between the occurrence of CMMLI (whether unilaterally or bilaterally) and the PP extension (*P*=0.12 for both) (Figure 5).

### 3.3. The Relationship between STB and PP

The relationship between the STB and the PP extension was studied as an additional hypothesis in this study but no statistically significant correlation was identified (*P*=0.14) (Table 3).

## 4. Discussion

This research studied the frequency of each form of STB and PP among orthodontic patients with and without CMMLI. STB and PP were present in 40% and 18.75% of the total study population, respectively. In both case and control groups, the frequency of the incomplete STB was higher than the complete type. Research has recently looked into the link between dentoskeletal anomalies and craniofacial and cervicovertebral anomalies [18, 22–26]. Consistent with the descriptive results of our study, several studies have reported that the prevalence of incomplete STB was higher than the complete form [19, 22, 27 to 29]. On the other hand, other studies have found that the incomplete form of PP was more prevalent than the complete type [22 and 28].

### 4.1. STB and CMMLI

According to the results of this study, the prevalence of STB was significantly greater in the patients with CMMLI than in the control group (52.5% vs. 27.5%), a finding similar to that of the previous studies [19, 28, and 29]. Although the prevalence of STB was higher in the patients with CMMLI than in the control group in the study of Ozturk et al., no significant relationship was found between STB and CMMLI. This finding was most likely due to the unbalanced distribution of males and females [22]. Other studies have found a significant relationship between STB with other dental abnormalities such as tooth displacement and aplasia [3, 21, 30–32]. According to the findings of many investigations, STB has been linked to a variety of disorders, including cleft lip and palate, Williams syndrome, severe craniofacial deviations, craniofacial classification, and palatal canine impactions [4, 18, 31, 33, and 34]. This could suggest the presence and role of neural crest cells and homeobox, or HOX genes during tooth formation and development [35].

### 4.2. PP and CMMLI

There is scant research studying the link between PP extension and dental abnormalities. The results of this study revealed a slightly greater prevalence of PP extension in patients with CMMLI compared to the control group (20% vs. 17.5%), although it was not statistically significant. In a study by Kaya et al. the prevalence of PP was found to be 26.6% and 16.9% among 75 patients with and 145 individuals without CMMLI, respectively. In contrast to the findings of the present study, the relationship between CMMLI and PP was significant, similar to the findings of Ozturk et al. [22 and 28]. Studies have demonstrated a significant relationship between PP and other dental anomalies, such as palatal impaction of maxillary canine, hypodontia, and hyperdontia [3, 4, 23, 35 to 37]. The importance of the neural crest as an embryonic source for cervical skeletal and dental development could explain this observation [3, 35].

### 4.3. Cervicovertebral Anomalies and Uni/Bilateral CMMLI

In addition to the preceding information, this study looked into the relationship between the STB and PP with the unilateral or bilateral occurrence of CMMLI. Except for the correlation between STB and bilateral CMMLI, the findings of this analysis revealed no significant relationship in any of the comparisons. To the best of authors' knowledge, only one other study looked into the relationship between bilateral CMMLI and Cervicovertebral anomalies [22]. Contrary to the results of this study, Ozturk et al. did not detect a significant relationship between bilateral CMMLI and STB, whereas they found a significant relationship between bilateral CMMLI and PP [22], which was in contrast to the current study's findings.

### 4.4. The Relationship between the STB and PP

Similar to the study by Leonardi et al. [35], there was no statistically significant association between STB and PP extension in this study. On the other hand, Tassoker et al. [38] discovered a significant relationship between STB and the prevalence of PP, which could be attributed to differences in the number of samples investigated. Another issue that might cause contradictory results is the quality of radiographs, leading to not identifying the anomalies, influenced by the proper selection of kVp and mA and the proper film processing method. Furthermore, heredity and external mechanical elements, such as carrying heavy objects on one's head and bone development due to aging can influence the study results.

### 4.5. STB and PP, Age and Gender

The investigations on the relationship between age/gender and STB/PP have yielded conflicting results. This study did not investigate the effects of age and gender on STB and PP. Research claim that there is no significant difference in STB and PP between men and women [4, 16, 39, and 40]. Alqahtani, on the other hand, found a significant association in CMMLI, indicating that males had more STB than females [19]. In addition, some studies have discovered a significant relationship between the dimensions of sella turcica in both men and women. The dimensions of sella turcica were also strongly related to the subjects' age [34, 41]. However, contrary to those findings, some research found no link between age and STB or PP [4, 27].

### 4.6. Clinical Significance

The relationship between STB and dental anomalies has been discussed in the literature and this study. The literature reported an increased frequency of PP development in individuals with dental anomalies such as CMMLI and palatally impacted canines [3, 19, 28, 29, and 35]. Moreover, some studies investigated the relationship between STB and skeletal malocclusions and found a higher frequency of STB in patients with skeletal Class II and III malocclusions compared to skeletal Class I malocclusion [1, 18, 42, 43]. As many cervicovertebral abnormalities such as STB and PP appear in early childhood, orthodontists must diagnose them on time. This can help predict dental and skeletal anomalies, leading clinicians to take preventative interventions. Orthodontists should be familiar with these types of malformations in the craniofacial area and not just focus on the maxillomandibular complex to have a comprehensive and broad overview contributing to a proper diagnosis and treatment plan.

### 4.7. Limitations

The use of two-dimensional lateral cephalometric films to examine a three-dimensional anatomical structure was a limitation of this work. Cone-beam computed tomography, which gives three-dimensional imaging, may be more accurate for this purpose. However, such imaging modalities are not recommended for routine usage in orthodontic patients due to high-dose radiation exposure [44]. Moreover, skeletal classification was not taken into account for participants with dental anomalies and equal sex distribution was overlooked in all groups. Therefore, future research should include people of similar ages and skeletal malocclusions, as well as an equal number of female and male participants (Table 4).

## 5. Conclusion

- Both STB and PP were found in individuals with CMMLI. The STB was positively correlated with CMMLI. Although the extension of PP was slightly more frequent in patients with CMMLI, no significant relationship was determined.
- The STB was significantly related to the occurrence of bilateral CMMLI but not to the occurrence of unilateral CMMLI. There was no significant difference between the PP extension and the occurrence of CMMLI (whether unilaterally or bilaterally).
- No significant relationship existed between the STB and the PP extension.

## Figures and Tables

**Table 1:** Age and gender distribution of study samples.

Variable	Gender *n* (%)		Age (years old)		
	Male	Female	Mean	Minimum	Maximum
Case	26 (32.5)	54 (67.5)	23.44	11	28
Control	32 (40)	48 (60)	21.39	11	28

**Table 2:** Frequency (%) of STB and PP among study groups.

Anomaly		Case		Control	Total	*P* value^*∗*^
		Unilateral	Bilateral
STB	Type I	22 (27.5)	16 (20)	58 (72.5)	96 (60)	0.01
	Type II	12 (15)	16 (20)	18 (22.5)	46 (28.75)	
	Type III	6 (7.5)	8 (10)	4 (5)	18 (11.25)	

PP	None	32 (40)	32 (40)	66 (82.5)	130 (81.25)	0.12
	Incomplete	0 (0)	4 (5)	4 (5)	8 (5)	
	Complete	8 (10)	4 (5)	10 (12.5)	22 (13.75)	

^*∗*^Chi-square analysis: *P* < 0.05 as significant.

**Table 3:** The relationship between STB and PP.

STB	Extension of PP		*P* value
	No	Yes	
No	72	24	0.14
Yes	58	6	

**Table 4:** List of abbreviations used in the text.

Abbreviation	Definition
CMMLIs	Congenital missing maxillary lateral incisors
DS	Dorsum sellae
PP	Ponticulus posticous
STB	Sella turcica bridging
TS	Tuberculum sella

## Data Availability

The datasets used and/or analyzed during the current study are available from the corresponding author upon reasonable request.

## References

[1] Meyer-Marcotty P., Reuther T., Stellzig-Eisenhauer A. (2009). Bridging of the sella turcica in skeletal Class III subjects. *The European Journal of Orthodontics*.

[2] Jankowski T., Jedliński M., Schmeidl K., Grocholewicz K., Janiszewska-Olszowska J. (2021). Sella turcica abnormalities, dental age and dental abnormalities in polish children. *International Journal of Environmental Research and Public Health*.

[3] Haji Ghadimi M., Amini F., Hamedi S., Rakhshan V. (2017). Associations among sella turcica bridging, atlas arcuate foramen (ponticulus posticus) development, atlas posterior arch deficiency, and the occurrence of palatally displaced canine impaction. *American Journal of Orthodontics and Dentofacial Orthopedics*.

[4] Dadgar S., Alimohamadi M., Rajabi N., Rakhshan V., Sobouti F. (2021). Associations among palatal impaction of canine, sella turcica bridging, and ponticulus posticus (atlas arcuate foramen). *Surgical and Radiologic Anatomy*.

[5] Minervini G., Romano A., Petruzzi M. (2018). Oral-facial-digital syndrome (OFD): 31-year follow-up management and monitoring. *Journal of Biological Regulators & Homeostatic Agents*.

[6] Chitroda P. K., Katti G., Baba I. A. (2013). Ponticulus posticus on the posterior arch of atlas, prevalence analysis in symptomatic and asymptomatic patients of gulbarga population. *Journal of Clinical and Diagnostic Research: Journal of Clinical and Diagnostic Research*.

[7] Elliott R. E., Tanweer O. (2014). The prevalence of the ponticulus posticus (arcuate foramen) and its importance in the Goel-Harms procedure: meta-analysis and review of the literature. *World Neurosurgery*.

[8] Al-Ani A. H., Antoun J. S., Thomson W. M., Merriman T. R., Farella M. (2017). Hypodontia: an update on its etiology, classification, and clinical management. *BioMed Research International*.

[9] Garib D. G., Alencar B. M., Lauris J. R. P., Baccetti T. (2010). Agenesis of maxillary lateral incisors and associated dental anomalies. *American Journal of Orthodontics and Dentofacial Orthopedics*.

[10] Rodrigues A. S., Teixeira E. C., Antunes L. S. (2020). Association between craniofacial morphological patterns and tooth agenesis-related genes. *Progress in Orthodontics*.

[11] Silva Meza R. (2003). Radiographic assessment of congenitally missing teeth in orthodontic patients. *International Journal of Paediatric Dentistry*.

[12] Sheikhi M., Sadeghi M. A., Ghorbanizadeh S. (2012). Prevalence of congenitally missing permanent teeth in Iran. *Dental Research Journal*.

[13] Rakhshan V. (2015). Congenitally missing teeth (hypodontia): a review of the literature concerning the etiology, prevalence, risk factors, patterns and treatment. *Dental Research Journal*.

[14] Kavadia S., Papadiochou S., Papadiochos I., Zafiriadis L. (2011). Agenesis of maxillary lateral incisors: a global overview of the clinical problem. *Orthodontics—The Art and Practice of Dentofacial Enhancement*.

[15] Sharma V., Chaudhary D., Mitra R. (2010). Prevalence of ponticulus posticus in Indian orthodontic patients. *Dentomaxillofacial Radiology*.

[16] Ali B., Shaikh A., Fida M. (2014). Association between sella turcica bridging and palatal canine impaction. *American Journal of Orthodontics and Dentofacial Orthopedics*.

[17] Najim A. A., Al-Nakib L. (2011). A cephalometric study of sella turcica size and morphology among young Iraqi normal population in comparison to patients with maxillary malposed canine. *Journal of Baghdad College of Dentistry*.

[18] Sobuti F., Dadgar S., Seifi A., Musavi S. J., Hadian H. (2018). Relationship between bridging and dimensions of sella turcica with classification of craniofacial skeleton. *Polish Journal of Radiology*.

[19] Alqahtani H. (2020). Association between sella turcica bridging and congenitally missing maxillary lateral incisors. *Journal of Dental Sciences*.

[20] Meriç P., Naoumova J. (2020). Web-based fully automated cephalometric analysis: comparisons between app-aided, computerized, and manual tracings. *Turkish Journal of Orthodontics*.

[21] Leonardi R., Barbato E., Vichi M., Caltabiano M. (2006). A sella turcica bridge in subjects with dental anomalies. *The European Journal of Orthodontics*.

[22] Ozturk T., Atilla A. O., Yagci A. (2020). Cervicovertebral anomalies and/or normal variants in patients with congenitally bilateral absent maxillary lateral incisors. *The Angle Orthodontist*.

[23] Adisen M. Z., Misirlioglu M. (2017). Prevalence of ponticulus posticus among patients with different dental malocclusions by digital lateral cephalogram: a comparative study. *Surgical and Radiologic Anatomy*.

[24] Amelinda V. P., Ismaniati N. A., Purbiati M. (2019). Association of Sella Turcica bridge and ponticulus posticus with palatally impacted canine and hypodontia. *Journal of International Dental and Medical Research*.

[25] Antonarakis G. S., Ghislanzoni L. H., Fisher D. M. (2021). Sella turcica bridging and tooth agenesis in children with unilateral cleft lip and palate. *The Cleft Palate-Craniofacial Journal*.

[26] Baidas L. F., Al-Kawari H. M., Al-Obaidan Z., Al-Marhoon A., Al-Shahrani S. (2018). Association of sella turcica bridging with palatal canine impaction in skeletal Class I and Class II. *Clinical, Cosmetic and Investigational Dentistry*.

[27] Karaman A., Cinarsoy Cigerim S., Kechagia N. (2021). Evaluation of the relationship between sella turcica bridging and dental anomalies. *Journal of Dentistry*.

[28] Kaya Y., Öztaş E., Goymen M., Keskin S. (2021). Sella turcica bridging and ponticulus posticus calcification in subjects with different dental anomalies. *American Journal of Orthodontics and Dentofacial Orthopedics*.

[29] Scribante A., Sfondrini M. F., Cassani M., Fraticelli D., Beccari S., Gandini P. (2017). Sella turcica bridging and dental anomalies: is there an association?. *International Journal of Paediatric Dentistry*.

[30] Axelsson S., Storhaug K., Kjær I. (2004). Post-natal size and morphology of the sella turcica in Williams syndrome. *The European Journal of Orthodontics*.

[31] Becktor J. P., Einersen S., Kjær I. (2000). A sella turcica bridge in subjects with severe craniofacial deviations. *The European Journal of Orthodontics*.

[32] Leonardi R., Farella M., Cobourne M. T. (2011). An association between sella turcica bridging and dental transposition. *The European Journal of Orthodontics*.

[33] Sinha S. P., Shetty A., Nayak U. K. (2020). The morphology of sella turcica in cleft and non-cleft individuals. *The Saudi Dental Journal*.

[34] Axelsson S., Storhaug K., Kjær I. (2004). Post-natal size and morphology of the sella turcica. Longitudinal cephalometric standards for Norwegians between 6 and 21 years of age. *The European Journal of Orthodontics*.

[35] Leonardi R., Barbato E., Vichi M., Caltabiano M. (2009). Skeletal anomalies and normal variants in patients with palatally displaced canines. *The Angle Orthodontist*.

[36] Pasini M., Giuca M. R., Ligori S. (2020). Association between Anatomical Variations and maxillary canine impaction: a retrospective study in orthodontics. *Applied Sciences*.

[37] Pérez I. E., Chávez A. K. (2015). Frequency of ponticulus posticus, sella turcica bridge and clinoid enlargement in cleft lip and palate Peruvian patients: a comparative study with non-cleft patients. *International Journal of Morphology*.

[38] Tassoker M., Kok H., Ozcan S. (2017). Investigation of the relationship between sella turcica bridge and ponticulus posticus: a lateral cephalometric study. *International Journal of Morphology*.

[39] Alkofide E. A. (2007). The shape and size of the sella turcica in skeletal Class I, Class II, and Class III Saudi subjects. *The European Journal of Orthodontics*.

[40] Kashio H., Toriya N., Osanai S. (2017). Prevalence and dimensions of sella turcica bridging in Japanese female orthodontic patients. *Orthodontic Waves*.

[41] Magat G., Ozcan Sener S. (2018). Morphometric analysis of the sella turcica in Turkish individuals with different dentofacial skeletal patterns. *Folia Morphologica*.

[42] Marşan G., Öztaş E. (2009). Incidence of bridging and dimensions of sella turcica in Class I and III Turkish adult female patients. *World Journal of Orthodontics*.

[43] Dasgupta P., Sen S., Srikanth H., Kamath G. (2018). Sella turcica bridging as a predictor of Class II malocclusion–an investigative study. *Journal of Stomatology, Oral and Maxillofacial Surgery*.

[44] Coşkun İ, Kaya B. (2018). Cone beam computed tomography in orthodontics. *Turkish Journal of Orthodontics*.

